# Replacing the wild type *loxP *site in BACs from the public domain with *lox66 *using a *lox66 *transposon

**DOI:** 10.1186/1756-0500-3-38

**Published:** 2010-02-19

**Authors:** Pradeep K Chatterjee, Leighcraft A Shakes, Naima Stennett, Vanessa L Richardson, Tennison L Malcolm, Ken R Harewood

**Affiliations:** 1 Department of Chemistry, North Carolina Central University, 1801 Fayetteville Street, Durham, NC 27707, USA; 2 Julius L Chambers Biomedical/Biotechnology Research Institute, North Carolina Central University, 1801 Fayetteville Street, Durham, NC 27707, USA; 3 Department of Biology, North Carolina Central University, 1801 Fayetteville Street, Durham, NC 27707, USA

## Abstract

**Background:**

Chromatin adjoining the site of integration of a transgene affects expression and renders comparisons of closely related transgenes, such as those derived from a BAC deletion series retrofitted with enhancer-traps, unreliable. Gene targeting to a pre-determined site on the chromosome is likely to alleviate the problem.

**Findings:**

A general procedure to replace the *loxP *site located at one end of genomic DNA inserts in BACs with *lox66 *is described. Truncating insert DNA from the *loxP *end with a Tn10 transposon carrying a *lox66 *site simultaneously substitutes the *loxP *with a *lox66 *sequence. The replacement occurs with high stringency, and the procedure should be applicable to all BACs in the public domain. Cre recombination of *loxP *with *lox66 *or *lox71 *was found to be as efficient as another *loxP *site during phage P1 transduction of small plasmids containing those sites. However the end-deletion of insert DNA in BACs using a *lox66 *transposon occurred at no more than 20% the efficiency observed with a *loxP *transposon. Differences in the ability of Cre protein available at different stages of the P1 life cycle to recombine identical versus non-identical *lox*-sites is likely responsible for this discrepancy. A possible mechanism to explain these findings is discussed.

**Conclusions:**

The *loxP/lox66 *replacement procedure should allow targeting BACs to a pre-positioned *lox71 *site in zebrafish chromosomes; a system where homologous recombination-mediated "knock-in" technology is unavailable.

## Findings

### Research Hypothesis

Expression of a transgene integrated into the germline of an animal is influenced by i) variation in copy number of transgene and ii) the effect of chromatin adjoining the site of integration [1]. Thus expression comparisons of closely related transgenes, such as those derived from a BAC deletion series retrofitted with enhancer-traps [2], become unreliable. Targeting transgenes to the same site on the chromosome alleviates the problem, and both "knock-in" technology using homologous recombination [1] and insertion of cDNA plasmids into a *loxP *site have been used in previous studies [3].

Because the *lox*-Cre recombination reaction/equilibrium favors excision, mutant sites such as *lox66 *and *lox71 *have been constructed to stably incorporate small plasmid DNA into chromosomes of plants and mouse ES cells [4,5]. BACs have not been used similarly, presumably because altering the *loxP *or *lox511 *sites flanking insert DNA in BACs is challenging. We describe a general procedure that readily overcomes this particular hurdle of converting a *loxP *to a *lox66 *in BACs.

### Materials and methods

Two BAC clones from the zebrafish genomic library, CH211-192O20, & CH211-43O16 designated here as BACs C & D respectively, were purchased from BAC/PAC resources, Oakland, California. These zebrafish BACs are in the pTARBAC2.1 vector. BACs C and D are of size 138.6 and 144.3 kb, respectively, as deduced from the location of their ends on the zebrafish chromosome 9 BAC contig of this region. They appear closer to 130 kb on FIGE. End-deletions of insert DNA in BACs were generated with *lox66 *transposons using procedures identical to those described earlier for *loxP *transposons [6,7]. Briefly, the *lox66 *transposon plasmid was introduced into the bacterial host containing the BAC using the calcium chloride transformation procedure. Transposition into BAC DNA was initiated by inducing the cells with IPTG. Cre recombinase was provided by infecting these cells with phage P1. The resulting end-deleted BACs were packaged as linear DNA in P1 heads, and used to infect fresh bacteria to regenerate the *lox66 *substituted BAC plasmids. Procedures for DNA isolation/purification from BAC deletions, FIGE analysis, end-sequencing of BAC deletions with transposon-based primers have been described earlier [8,9]. Identical procedures were also followed with the BAC deletions generated with *lox66 *enhancer-trap transposons. Primers used for sequencing the newly created end of BACs are:

**Seq 1**......5' d GACAAGATGTGTATCCACCTTAAC 3'

**Seq 4-compliment**......5' d CCGTTTTTATCAGGCTCTGGGAG 3'

**LF8-compliment**......5' d CTTGATTCCATTCATCTGTAGTG 3'

## Results

### Cre-recombination of mutant *lox *sites

**Figure 1:**
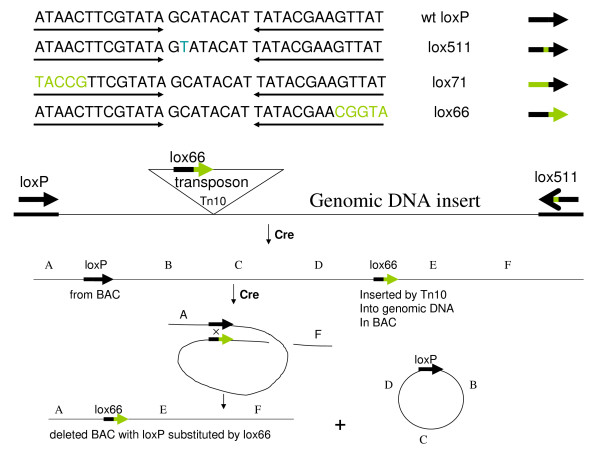
**Schematic diagram for replacing *loxP *with *lox66 *in BACs**. A set of mutant lox sites is shown in the top panel, with the mutant nucleotides shown in green. The inverted repeats are underlined. The bottom panel sketches our strategy to convert a wild type *loxP *to a *lox66 *mutant site in BACs while making end-deletions of genomic DNA insert from *loxP *end.

Mutant *lox *sites generated over the years [4,5,10-15] are of two categories: i) mutations in the 8 bp asymmetric spacer and ii) mutations in the 13 bp inverted repeats (Figure 1). The 8 bp spacer region is thought to form Holiday-like structure with perfect base pairing required during the recombination process. Single nucleotide differences in spacer of two *lox *sites render recombination between them very inefficient [10-13,16]. Nevertheless varying degrees of promiscuity in recombining different spacer-mutant *lox *sites have been reported; using both partially purified Cre-extracts *in vitro *[10,11,14,15], and Cre over-expressed in cells [12,13,15,17]. High levels of stringency *in vivo *can be achieved however with Cre protein expressed from its native source namely, a phage P1 infection [7,16].

Nucleotide substitutions in the 13 bp inverted repeats (palindromic arms) appear not to severely limit its recombination with a wild type *loxP *sequence, and this tolerance increases for substitutions towards the outer ends of these inverted repeats [18]. Cre protein binds to these 13 bp palindromic arms to recombine two *lox *sites [18,19], and the protein-protein interactions between Cre molecules appear to compensate for possible distortions in Cre binding to mutant-arm DNA.

Wild-type *loxP *and the arm-mutant *lox66 *have identical spacers, and recombine well with Cre [5]. We used this rationale to substitute a *lox66 *for the wild-type *loxP *site in BACs while truncating the insert DNA using a *lox66 *transposon (Figure 1).

### Stringency in Cre recombinations between wild type and mutant *lox*-sites

While cross recombination between *loxP *and *lox511 *had been reported to occur at efficiencies ranging from 5 to 100% in a variety of settings that expressed Cre constitutively [11-13,15,17], it was determined to be no more than 0.5% in our BAC end-deletion procedures where Cre protein is generated by a phage P1 infection [7,16]. The possibility that phage P1 infection-derived Cre protein in our procedures might prove too stringent to allow efficient recombination between *loxP *and *lox66*, despite the fact that *lox66 *is an arm mutant and not a spacer mutant like *lox511*, was explored first by testing the ability of phage P1, carrying wild type *loxP*, to transduce a plasmid containing a *lox66 *site [16].

### Testing Cross Recombination between *loxP *of phage P1 & mutant *lox sites *in plasmids carrying antibiotic resistance genes with phage P1-derived Cre protein

*E. Coli *with small plasmids carrying one of several mutant *lox *sites and a gene conferring resistance to an antibiotic were each infected with phage P1. The P1 lysates were used to infect fresh bacteria, and plated on LB agar containing the antibiotic whose resistance gene was carried by the plasmid [16]. The results are shown in Table 1.

**Table 1:** Phage P1 transduction of plasmids with mutant lox sites

Clone	Marker	Lox Site	Transduction with phage P1
a	ampR	lox66	++++
b	ampR	lox66	++++
			
c	ampR	lox511	0
			
d	ampR	wt loxP	++++
			
g	ampR	lox71	++++
h	ampR	lox71	++++
			
i	camR	lox5171	0
			
j	camR	lox2272	0
			
k	kanR	wt loxP	++++
l	kanR	wt loxP	++++

0-2 colonies/plate: 0

500-1000 colonies/plate: ++++

The results indicate that phage P1 can transduce a *lox66 *plasmid just as efficiently as a wild type *loxP *plasmid (compare rows 1 & 2 with 4 in Table 1). However, phage P1 was unable to transduce plasmids with the spacer mutants *lox511*, *lox5171 *and *lox2272 *(mutants described in references 10 and 11), and these were used as negative controls in the experiment (rows 3, 7 & 8). The data shown are from two independent experiments. Note that both the *lox *"arm mutants", *lox66 *and *lox71*, are efficiently transduced, while all of the "spacer mutants", *lox511*, *lox2272 *and *lox5171 *are not (compare rows 1, 2, 5, 6 with 3, 7, 8). There was a quantitative difference between *lox511 *and *lox5171 *or *lox2272*: while the latter two produced zero colonies on both runs, the *lox511 *produced 2 and 4 colonies in the two experiments. The results are in line with earlier findings [16,7,5], although the high efficiency of transducing *lox66 *and *lox71 *plasmids by wild type *loxP *in phage P1 under our more stringent *lox*-Cre recombination conditions was surprising.

**Figure 2:**
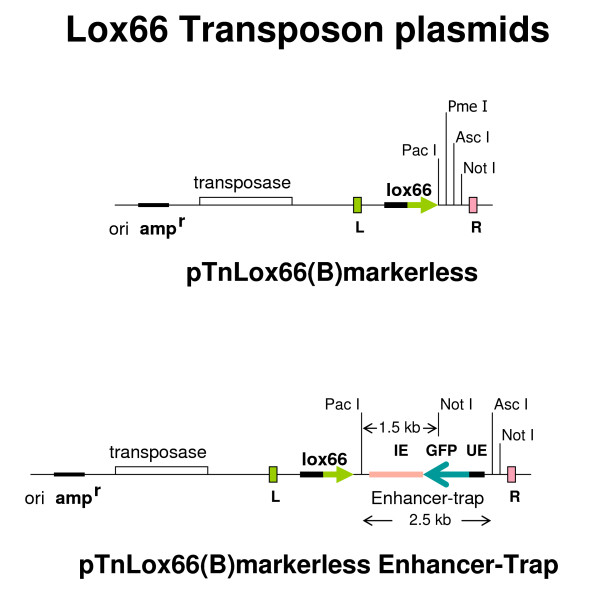
**Schematic representation of the two *lox66 *transposons constructed**. Both use the same framework of markerless transposons with *loxP *or lox511 [20,7]. The enhancer-trap is adapted from [2].

Having demonstrated efficient cross recombination between *lox66 *and *loxP *using phage P1 derived Cre protein, we tested the *lox66 *transposons to generate end-deletions of insert DNA in BACs. The following *lox66 *markerless transposon plasmids, similar to our other markerless transposons reported earlier [20,7], were constructed (Figure 2).

### Progressive end-deletions of insert DNA in BACs with pTnLox66(B)markerless and pTnLox66(B)markerless Enhancer-Trap transposons

**Figure 3:**
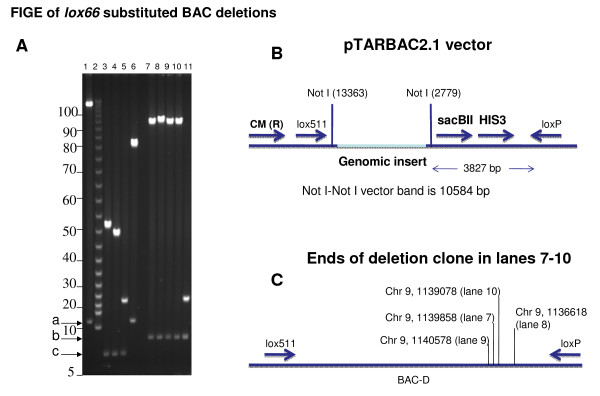
**FIGE analysis of BAC DNA isolated from deletions generated with *lox66 *transposons**: **Panel A**: The BAC DNA was digested with Not I prior to FIGE. Lane 1 shows DNA from starting BAC-C. Lanes 3-5 and 7-11 display DNA from BAC deletions generated by Cre-recombination of *lox66 *with *loxP *using transposons pTnLox66(B)markerless and pTnLox66(B)markerless Enhancer-Trap transposon, respectively. Lane 6 shows DNA from a *loxP*-Cre independent internal deletion. Lane 2 shows a 5 kb ladder. The vector DNA bands generated with Not I **a, b, c**, are indicated by the arrows to the left. Size of vector bands **b **and **c **are consistent with *loxP*-Cre dependent recombinations, while vector band **a **arises from starting BAC-C or from an internal deletion in BAC-C. DNA from BAC deletions made with pTnLox66(B)markerless transposon generated the Not I vector band of the expected size (~6.6 kb) shown in lanes 3-5 of Figure 3 (marked by arrow **c**). The BAC deletion shown in lane 6 arises from an internal deletion in the genomic insert DNA, and is independent of *lox*-Cre recombination. The vector DNA band upon Not I digestion of this clone is 10.6 kb in size, and is identical to that of starting BAC clone C (displayed in lane 1, Figure 3A and marked by arrow **a**). This vector DNA band serves as a characteristic identifying feature for internal deletions [20], and can comprise ~90% of isolates in end-deletions made with certain BAC clones (PKC unpublished observations). These arise due to recombinogenic sites in insert DNA (discussed in [20]). **Panel B**: A schematic representation of the Not I sites in pTARBAC2.1 vector DNA in BAC clones is shown. **Panel C**: Location of ends of *lox66 *substituted deletion clones in lanes7-10 on zebrafish chromosome 9 is indicated. These were obtained by BLAST analyses of the BAC end sequences derived with Seq 1 primer [Additional File 1] with the zebrafish genome sequence.

End deletions of genomic DNA in BAC clone C was made with pTnLox66(B)markerless transposon (Figure 2, top panel) exactly as described earlier with *loxP *markerless transposons [20,7,2]. DNA isolated from a set of deletions is shown in lanes 3-11, Figure 3A. Note that inversions of BAC DNA resulting from transpositions of *lox66 *in the opposite orientation to that in the BAC vector (schematic in Figure 3B) are not recovered because the starting BAC clones are larger than P1-headful length [20].

End-deletions of insert DNA in a different BAC clone D with pTnLox66(B)markerless Enhancer-Trap transposon (schematic in lower panel of Figure 2) is displayed in lanes 7-11 of Figure 3, panel A. Not I sites in pTARBAC2.1 vector DNA in BAC clones is shown in panel B. Note that the 3827 bp of DNA between *loxP *and the Not I site at 2779 is replaced during recombination of transposed *lox66 *and *loxP *in BAC vector. Thus in deletions with the pTnLox66(B)markerless transposon the BAC vector band obtained with Not I digestion is ~6.6 kb (see lanes 3-5 of Figure 3A). Deletions with pTnLox66(B)markerless Enhancer-Trap transposon results in a Not I vector DNA band larger by 1.5 kb, (lanes 7-11 Figure 3A), due to the Not I site being further away in the enhancer-trap (see lower panel of Figure 2). BAC insert DNA sequencing with the transposon based primer Seq 1 [9] indicates DNA size in deletions shown in lanes 7-10 are all slightly different despite their inadequate resolution in the FIGE (Figure 3A). This is indicated in the schematic diagram in panel C of Figure 3, and the actual sequences included in [Additional File 1].

Progressive end-deletions using the *lox66 *transposons were generated at an overall efficiency of 10-20% of that obtained with the wild type *loxP *transposons, depending on whether other recombinogenic sites existed in the genomic DNA insert of the BAC clone. For example, efficiency was at the lower end of ~10% with BAC clone C; which consistently generates a large percentage of *lox*-Cre independent internal deletions. As much as ~40% of clones isolated comprise internal deletions of that particular size shown in lane 6, Figure 3A, when a wild type *loxP*markerless transposon is used. Note that *lox*-Cre independent internal deletions of genomic insert are readily isolated here because there is no selection for transposition itself in these markerless transposons, only selection for deleting insert DNA to less than P1 headful packaging capacity (see [20] for discussion).

End-deletions with the *lox66 *transposons approached ~20% with a different BAC clone D, containing APPb gene sequences from a different region of the chromosome. BAC-D produces far fewer internal deletions (not shown). The lower efficiency of generating end-deletions with either of the two *lox66 *transposons is in sharp contrast to the transduction experiments with phage P1, displayed in Table 1, where no difference was observed between wild type *loxP *and *lox66 *carrying plasmids.

### Sequencing newly created end of *lox66 *BAC deletions in both directions with transposon based primers

**Figure 4:**
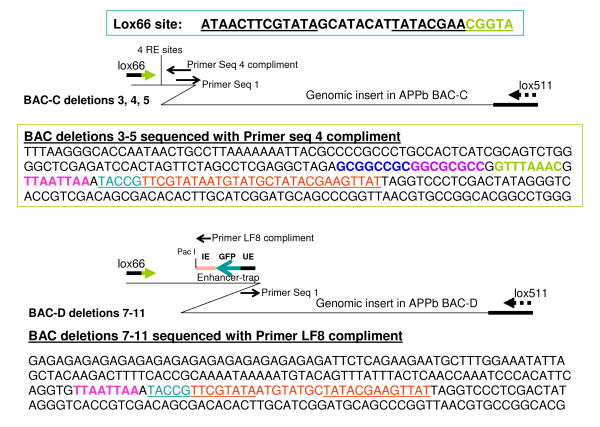
**BAC deletions generated with *lox66 *transposons pTnLox66(B)markerless and pTnLox66(B)markerless Enhancer-Trap sequenced with Primers Seq 4-compliment and LF8-compliment respectively**. **Top Panel**: Sequence of BAC-C deletions 3, 4, 5, is shown. The four restriction enzyme sites (4 RE sites) indicated in the schematic representation are Not I, Asc I, Pme I and Pac I, and correspond to sites in pTnLox66(B)markerless transposon shown in Figure 2 top panel. These sites are highlighted by different colors in the sequence presented. **Bottom Panel**: Sequence of BAC-D deletions 7-11 obtained with pTnLox66(B)markerless Enhancer-Trap transposon is shown. The Pac I site is highlighted in color. Sequence complimentary to the *lox66 *site is colored and underlined in both panels. Sequencing in the opposite direction with transposon end-based primer Seq 1 in deletion clones 3-5 and 7-11 indicates zebrafish DNA which BLASTs to chromosome 9 (not shown). The locations of these sequences on Chr 9 are consistent with end-points of the BAC deletions expected from their sizes on the FIGE gel shown in Figure 3.

The DNA from BAC deletions generated with the two *lox66 *transposons was sequenced with primers complementary to the transposon end (see [9] for details). These are seq 1, and seq 4-compliment for deletions made with pTnLox66(B)markerless transposon, and seq 1 and LF8-compliment for deletions made with pTnLox66(B)markerless Enhancer-Trap transposon. While primers Seq 1 and Seq 4 have been described earlier [9], primer LF8-compliment was designed from within the intron enhancer of Amyloid Precursor Protein (APPb) gene [2]. The results are shown schematically in Figure 4.

The Seq 1 primer sits 38 nucleotides from the transposon end, and sequences generated with it read outward into the genomic insert DNA (9). Sequencing BAC deletions displayed in lanes 3-5 and 7-11 of Figure 3A with Seq 1 primer indicate that progressive truncations have occurred from the *loxP *end of genomic insert DNA in each of these clones from starting BACs C and D respectively. BLAST analyses of these sequences determine the deletion end points (shown in Figure 3C), and are consistent with their mobility on FIGE.

Sequences of BAC deletions obtained using either Seq 4-compliment or LF8-compliment read in the opposite direction, as indicated in Figure 4. The results clearly demonstrate that the *loxP *site in the parent BACs have been replaced with *lox66*. The sequences in Figure 4 also indicate that restriction enzyme sites in the starting transposon have been preserved in the BAC deletions (compare restriction sites in Figures 2 and 4).

## Discussion

We describe a general approach to replace the wild type *loxP *sequence located at one end of genomic DNA inserts in all public domain BACs with a *lox66 *site. The procedure uses a *lox66 *transposon to trim insert DNA from the *loxP *end and simultaneously replace the original *loxP *with a *lox66 *site. Replacement of *loxP *with *lox66 *occurs with high fidelity. Although the genomic DNA insert is truncated in the process, the size of insert DNA remaining in the *lox66 *BAC can be as large as 105 kb, the limit encountered by the ~110 kb packaging capacity of the P1 phage head. The resulting 105 kb insert DNA size, the remainder being BAC vector, is unlikely to be a drawback in most applications; because a majority of vertebrate genes can be housed in their entirety within this size limit: more than half of non-coding gene-regulatory sequences in vertebrates are located within this span of DNA adjoining start sites of genes [21]. Therefore *lox66 *BACs housing entire genes in their chromosomal contexts can now be stably integrated to pre-positioned *lox71 *sites in chromosomes using Cre recombination as described earlier for small plasmids [4,5].

The fidelity of substituting *lox66 *for *loxP *in BACs is high: No truncations occurred from the *lox511 *side of genomic DNA insert in our experiments using the *lox66 *transposons. This should have been easy to detect because a different sized vector DNA band would have been generated upon Not I digestion of the DNA (see Figure 3B). The high fidelity of recombination observed in the *loxP *site substitutions is consistent with the transduction experiments described here (compare rows 1, 2 and 5, 6 with row 3 in Table 1) and with earlier studies using P1 phage-generated Cre protein [7,16].

The results shown in Figures 3 and 4 indicate that additional changes, such as incorporating enhancer-traps [2] or other reporter and/or selectable marker genes can readily be made in the *lox66*-BACs during the substitution process. The methodology should facilitate targeting functionalized *lox66*-BACs to a pre-positioned *lox71 *site on the chromosome to generate transgenic animals. The approach should be of special interest in systems, such as zebrafish, where "knock-in" technology using homologous recombination are un-available due to genome duplication in an ancestral teleost [22]. Targeted integration using this strategy has been reported recently in zebrafish; but only to integrate small *lox66 *plasmids to *lox71 *sites on chromosomes [23].

Targeting *loxP *plasmids to vertebrate genomes can be affected both by the DNA topoisomerase activity of Cre protein and cryptic *lox *sites in chromosomes [24-27]. The topoisomerase activity of Cre is unavoidable, and might explain the low efficiency of integrating small *lox66 *plasmids to chromosomal *lox71 *sites in zebrafish [23]. Cryptic *lox *sites are also likely to reduce efficiency, although integration at those sites is expected to be less efficient than at authentic *lox71 *sites. Despite these potential complications, targeting of small *lox66 *plasmids to a *lox71 *site has been successful in zebrafish [23], plants [4] and mouse ES cells [5,3], and therefore *lox66 *BACs should be targetable in a similar manner.

We believe using a *lox66 *transposon to substitute the *loxP *site in BACs should be easier than using homologous recombination in *E.coli *because the same *lox66 *transposon can be used with all BACs in the public domain. Recombineering approaches on the other hand require building at least one arm of homology for each BAC [28-30]. Additional alterations to the BAC are easy to incorporate with *lox66 *transposons, but difficult otherwise.

**Figure 5:**
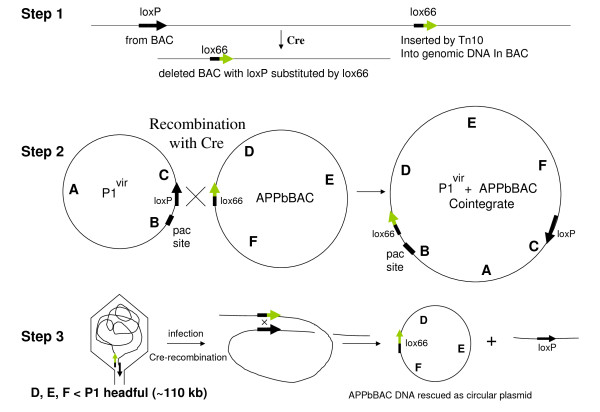
**A schematic representation of the three Cre-recombination steps in the end-deletion/*lox66 *substitution process**. Step 1 shows the end-deletion of genomic insert DNA by the transposed *lox66 *site recombining with the *loxP *endogenous to the BAC clone. Step 2 illustrates co-integrate formation between *lox66 *APPb BAC and P1 phage DNA carrying *loxP*. Step 3 shows regeneration of circular *lox66 *BAC from the linear DNA in the phage head after infection of fresh bacteria. The *lox511 *site at the other end of genomic insert DNA is omitted for clarity purposes, as it does not play any role in Cre recombinations with either *lox66 *or *loxP*. Note that the relative position of the two *lox *sites in the linear DNA inside the phage head determines whether *lox66 *or *loxP *is retained in the BAC. This in turn is determined by the location of the "pac site" in the co-integrate shown in Step 2 (see [16,20], for detailed discussion).

The overall efficiency of making end-deletions with the *lox66 *transposons is 5-10 fold lower compared to *loxP *transposons, despite there being no difference between the two in phage P1 transduction of plasmids containing either site. This was puzzling. The BAC end-deletion procedure can be broken down into three discrete Cre-mediated recombination steps (shown schematically in Figure 5), namely 1) creating the *lox66*-*loxP *deletion in BAC after the *lox66 *site transposition, 2) generating the co-integrate between deleted *lox66-*BAC and P1 phage, and 3) circularizing the linear BAC DNA packaged in the phage head upon re-entering fresh bacteria (see references 16, 20 for details). Each of these steps requires Cre recombination between *loxP *and *lox66 *sites. While steps 2) and 3) are common to both P1 transductions and BAC end-deletion procedures, step 1) is unique to the latter. Note also that steps 1) and 3) most likely occur during the early stages of the P1 infection, with lower levels of Cre protein around, while step 2) occurs late in infection with probably higher levels of Cre protein in the cell. It is tempting to speculate therefore that step 1) is likely to be more stringent than step 2), and might discriminate between *loxP *and *lox66 *sites so as to slightly disfavor the *loxP-lox66 *compared to a *loxP-loxP *recombination. Thus fewer truncations are likely to occur with *lox66 *inserted into the genomic DNA; resulting in fewer BAC inserts capable of packaging both the *lox66 *and *loxP *sites within the same phage head that ultimately allows them to be circularized in the next round of infection [16,20]. Such rationalization would also require that a similar discrimination in step 3) is not enough to lower survival of the truncated linear BAC DNA flanked by *loxP-lox66 *within phage P1 heads through circularization. A delay in degradation of the linear DNA upon entry into the cell might be sufficient to overcome this discrimination. Also other recombinases in the cell might help out in the process of circularization, as noted earlier [16]. Loss of stringency of *lox*-Cre recombinations with higher levels of Cre protein, proposed for step 2), is most likely responsible for the wide range of promiscuity observed in previous studies where Cre was expressed constitutively in cells or used *in vitro *[11-15,17].

Several interesting conclusions can be drawn from considering the asymmetry of the *lox*-Cre recombination reactions involved in the substitution process. There is directionality to the *loxP *site arising from the 8-base asymmetric spacer. Cre-recombination of identical spacer *lox *sites occur readily, mutant or otherwise, while those between non-identical spacers is severely restricted [16,7]. Thus substitution of the *lox511 *site at the other end of insert DNA in BACs with *lox66 *is likely feasible when it also carries the same spacer mutation as *lox511*. The directionality of the *lox*-Cre recombination should also prevent substitution of *lox71 *for *loxP *in BACs: because the arm mutation in *lox71 *is on the rear end of the arrow shown in Figure 1 (top panel), the Cre recombination after transposition of a *lox71 *site into genomic DNA would result in *lox71 *ending up in the deleted portion of the genomic insert (see bottom panel of Figure 1). However, transduction of plasmids carrying *lox71 *by phage P1 is not affected by this asymmetry: the *lox71 *site should end up in segment F-C of cointegrate rather than segment B-D as in the case of *lox66 *(refer to Step 2 of Figure 5). The linear DNA in the P1 phage head should then be flanked by *loxP *at left and *lox71 *on right, respectively (Step 3 of Figure 5). Upon Cre-recombination this DNA should be able to circularize with *lox71 *in the BAC and *loxP *in the small linear piece of DNA from the termini (step 3 Figure 5).

The lower efficiency of end-deletions with *lox66 *compared to *loxP *transposons should not pose a hurdle because several thousand deletion clones are generated in each deletion/substitution experiment, and one can screen a sufficient number of clones to obtain the desired number of *lox66 *substituted BAC deletions. Using this end-deletion/substitution procedure, BACs as large as 110 kb with *loxP *replaced by *lox66 *can be generated with minimal effort using the same *lox66 *transposon for all BACs in the public domain.

## Abbreviations

BAC: bacterial artificial chromosome; FIGE: Field inversion gel electrophoresis; APPb: Amyloid Precursor Protein.

## Competing interests

The authors declare that they have no competing interests.

## Authors' contributions

PKC carried out the design of experiments, *lox66 *transposon retrofitting of BACs, analyses of data and writing of manuscript. LAS, NS, VLR and TLM screened BAC deletion libraries using FIGE, end-sequencing of *lox66 *substituted BACs and bio-informatic analyses of data. KRH helped with writing, provided critical evaluation of scientific content of manuscript and helped with funds. All authors read and approved the final manuscript.

## Supplementary Material

Additional file 1 **Sequences of BAC deletion ends**. Sequences of the end points of the BAC deletions shown in lanes 7-10, Figure 3, on zebrafish chromosome 9. These were obtained by direct BAC end-sequencing with the Seq 1 primer located in the transposon end retained in the deletion clone. Click here for file

## References

[B1] Bronson S Plaehn E Kluckman K Hagaman J Maeda N Smithies O Single-copy transgenic mice with chosen-site integration Proc Natl Acad Sci USA 1996 93 9067 9072 10.1073/pnas.93.17.9067 8799155 PMC38596

[B2] Shakes LA Malcolm TL Allen KL De S Harewood KR Chatterjee PK Context dependent function of APPb Enhancer identified using Enhancer Trap-containing BACs as Transgenes in Zebrafish Nucleic Acids Research 2008 36 6237 6248 10.1093/nar/gkn628 18832376 PMC2577333

[B3] Sauer B Hendenson N Targeted insertion of exogenous DNA into the eukaryotic genome by the Cre recombinase New Biol 1990 441 449 2288914

[B4] Albert H Dale EC Lee E Ow DW Site-specific integration of DNA into wild-type and mutant *lox *sites placed in the plant genome Plant J 1995 7 649 659 10.1046/j.1365-313X.1995.7040649.x 7742860

[B5] Araki K Araki M Yamamura K Targeted integration of DNA using mutant lox sites in embryonic stem cells Nucleic Acids Res 1997 25 868 872 10.1093/nar/25.4.868 9016639 PMC146486

[B6] Chatterjee PK Retrofitting BACs and PACs with LoxP Transposons to Generate Nested Deletions. "Bacterial Artificial Chromosomes" Methods in Mol Biology 2004 1 231 241 10.1385/1-59259-752-1:231 15020829

[B7] Shakes LA Garland DM Srivastava DK Harewood KR Chatterjee PK Minimal Cross-recombination between wild type and lox511 sites *in vivo *facilitates Truncating Both Ends of Large DNA Inserts in pBACe3.6 and Related Vectors Nucleic Acids Research 2005 33 e118 10.1093/nar/gni119 16061933 PMC1182172

[B8] Chatterjee PK Baker JC Jr Shaying Zhao, Marvin Stodolsky Preparing Nested Deletions Template DNA for Field Inversion Gel Electrophoresis Analyses and Position-Specific End Sequencing With Transposon Primers. "Bacterial Artificial Chromosomes" vol 1, pp 243-254 Methods in Mol Biology series 2004 255 The Humana Press Inc 10.1385/1-59259-752-1:243 15020830

[B9] Chatterjee PK Yarnall DP Haneline SA Godlevski MM Thornber SJ Robinson PS Davies HE White NJ Riley JH Shepherd NS Direct Sequencing of Bacterial and P1 Artificial Chromosome Nested-deletions for Identifying Position-Specific Single Nucleotide Polymorphisms Proc Natl Acad Sci (USA) 1999 96 13276 13281 10.1073/pnas.96.23.13276 PMC23938 10557311

[B10] Hoess RH Wierzbicki A Abremski K The role of the *loxP *spacer region in P1 site-specific recombination Nucleic Acids Res 1986 14 2287 2300 10.1093/nar/14.5.2287 3457367 PMC339658

[B11] Lee G Saito I Role of nucleotide sequences of *loxP *spacer region in Cre-mediated recombination Gene 1998 216 55 65 10.1016/S0378-1119(98)00325-4 9714735

[B12] Siegel RW Jain R Bradbury A Using an *in vivo *phagemid system to identify non-compatible *loxP *sequences FEBS lett 2001 505 467 473 10.1016/S0014-5793(01)02806-X 11576551

[B13] Langer SJ Ghafoori AP Byrd M Leinwand L A genetic screen identifies novel non-compatible loxP sites Nucleic Acids Res 2002 30 3067 3077 10.1093/nar/gkf421 12136089 PMC135742

[B14] Missirlis PI Smailus DE Holt RA A high-throughput screen identifying sequence and promiscuity characteristics of the *lox *P spacer region in Cre-mediated recombination BMC Genomics 2006 7 73 85 10.1186/1471-2164-7-73 16595017 PMC1479339

[B15] Sheren J Langer SJ Leinwand LA A randomized library approach to identifying functional lox site domains for the Cre recombinase Nucleic Acids Res 2007 35 5464 5473 10.1093/nar/gkm604 17702764 PMC2018622

[B16] Chatterjee PK Shakes LA Srivastava DK Garland DM Harewood KR Moore KJ Coren JS Mutually exclusive recombination of wild-type and mutant loxP sites *in vivo *facilitates transposon-mediated deletions from both ends of genomic DNA in PACs Nucleic Acids Res 2004 32 5668 5676 10.1093/nar/gkh900 15494454 PMC524307

[B17] Wang Z Engler P Longacre A Storb U An efficient method for high-fidelity BAC/PAC retrofitting with a selectable marker for mammalian cell transfection Genome Res 2001 11 137 142 10.1101/gr.159001 11156622 PMC311050

[B18] Hartung M Kisters-Woike B Cre mutants with altered DNA binding properties J Biol Chem 1998 273 22884 22891 10.1074/jbc.273.36.22884 9722507

[B19] Guo F Gopaul DN Van Duyne GD Structure of Cre recombinase complexed with DNA in a site-specific recombination synapse Nature 1997 389 40 46 10.1038/37925 9288963

[B20] Chatterjee PK Mukherjee S Shakes LA Wilson W III Harewood KR Byrd G Selecting Transpositions of a Markerless Transposon Using Phage P1 Headful Packaging: New Transposons for Functionally Mapping Long Range Regulatory Sequences in BACs Analytical Biochemistry 2004 335 305 315 10.1016/j.ab.2004.09.016 15556570

[B21] Woolfe A Goodson M Goode DK Snell P McEwen GK Vavouri T Smith SF North P Callaway H Kelly K Walter K Abnizova I Gilks W Edwards YJ Cooke JE Elgar G Highly conserved non-coding sequences are associated with vertebrate development PLoS Biol 2005 3 e7 10.1371/journal.pbio.0030007 15630479 PMC526512

[B22] Kleinjan DA Bancewicz RM Gautier P Dahm R Schonthaler HB Damante G Seawright A Hever AM Yeyati PL van Heyningen V Coutinho P Subfunctionalization of duplicated zebrafish pax6 genes by cis-regulatory divergence PLoS Genet 2008 4 e29 10.1371/journal.pgen.0040029 18282108 PMC2242813

[B23] Liu WY Wang Y Qin Y Wang YP Zhu ZY Site-Directed Gene Integration in Transgenic Zebrafish Mediated by Cre Recombinase Using a Combination of Mutant Lox Sites Marine Biotechnology 2007 9 420 428 10.1007/s10126-007-9000-x 17503154

[B24] Abremski K Wierzbicki A Frommer B Hoess RH Bacteriophage P1 Cre-loxP site-specific recombination. Site-specific DNA topoisomerase activity of the Cre recombination protein J Biol Chem 1986 261 391 396 3001054

[B25] Thyagarajan B Guimaraes MJ Groth AC Calos MP Mammalian genomes contain active recombinase recognition sites Gene 2000 244 47 54 10.1016/S0378-1119(00)00008-1 10689186

[B26] Schmidt EE Taylor DS Prigge JR Barnett S Capecchi MR Illegitimate Cre-dependent chromosome rearrangements in transgenic mouse spermatids Proc Natl Acad Sci USA 2000 97 13702 13707 10.1073/pnas.240471297 11087830 PMC17639

[B27] Semprini S Troup TJ Kotelevtseva N King K Davis JR Mullins LJ Chapman KE Dunbar DR Mullins JJ Cryptic loxP sites in mammalian genomes: genome-wide distribution and relevance for the efficiency of BAC/PAC recombineering techniques Nucleic Acids Res 2007 35 1402 1410 10.1093/nar/gkl1108 17284462 PMC1865043

[B28] Gong S Yang XW Li C Heintz N Highly efficient modification of bacterial artificial chromosomes (BACs) using novel shuttle vectors containing the R6Kgamma origin of replication Genome Res 2002 12 1992 1998 10.1101/gr.476202 12466304 PMC187570

[B29] Muyrers JP Zhang Y Testa G Stewart AF Rapid modification of Bacterial Artificial Chromosomes by ET recombination Nucleic Acids Res 1999 27 1555 1557 10.1093/nar/27.6.1555 10037821 PMC148353

[B30] Jessen JR Meng A McFarlane RJ Paw BH Zon LI Smith GR Lin S Modification of bacterial artificial chromosomes through chi-stimulated homologous recombination and its application in zebrafish transgenesis Proc Natl Acad Sci USA 1998 95 5121 5126 10.1073/pnas.95.9.5121 9560239 PMC20224

