# Supplementary material for: Replacing the wild type loxP site in BACs from the public domain with lox66 using a lox66 transposon
Source: BMC Res Notes. 2010 Feb 19;3:38. doi: 10.1186/1756-0500-3-38 (PMC2841073; doi:10.1186/1756-0500-3-38)
Supplement: Additional file 1 — Sequences of BAC deletion ends. Sequences of the end points of the BAC deletions shown in lanes 7-10, Figure 3, on zebrafish chromosome 9. These were obtained by direct BAC end-sequencing with the Seq 1 primer located in the transposon end retained in the deletion clone. [file 1756-0500-3-38-S1.DOCX]

**SUPPLEMENTARY FILE:**

**End-Sequences of *lox66* substituted BAC deletions obtained with primer Seq 1**

**Clone in Lane 9: BLASTs to zebrafish Chr 9, location 1140578**

TACGAGATCAGATAGTTCTCGATAACTTCCCAAGAATAGACCGATAACTGCGCTAATTTG

TACATAATAGGAGCTTGTGACTTTAACTTTAGTCGCATGTTTTTTGAGTTGGAGGAAACC

GGAATACCCGGGGAAAACCCACACGAACACGGGGAGAACATGCAAACTCAGCAAAGAGAG

TGTCAGCTGGCTCAGCTAGTGTTTGAACCAACGACCCCCTTGCTGTTGGGCAACAGTGAT

AGCCACTGTGTCACCCGAACTTTGAATTGAGGAGGAGGGAGAAGGGATGGATGGGGGGGG

TAACGAAGATGAGGAATGAAGTTTAGGCTGACTATTTACATTAAATTTAGAATGATTCGA

TTGGCTAGTTAGTGATTAGTGTTAGGGATCAGCTGTAGTGAATCCCATCACGTGCTCCTC

TCAAAATTAGTTTGCGAAACTTCCCTTAATATTTTTTTATGAATTCTTGAAATATATTAT

CTTAATGTCTATAATAAATTAAATCTTAGGTGTCCGTTGGAAATCTCACTCAATTCATCA

GAACATTTACAATCTCCATGACTGAACTGCAAGTTATCAATATCAGTATTATAAATAAAA

**Clone in Lane 7: BLASTs to zebrafish Chr 9, location 1139858**

GCTTGTGTCTGTTTCGAGTGCGCCCGCATGTCAGTGTGTGTGTTTGTGAATCTGCTGAGC

ACTGCAGTACTGGATGTGCCTCTCTCTCTGGGCGTGTGTGTTTATCTCAGTGTGTGGCCA

GTGATAGAGCTGCAGTCGGCCTCTGGTCAGACCGGTCTGTGGCAGTGGTTCAGTGAGCGC

TTTCTGCTCGGTTAAGCACAATGCTGGACTCTGTGGGAAAGTGACAGCGGAGCAGCCCTC

ATCCTGACAGGGCATATTCCTCACAGCCGGGCTGGCTGTTCCCGCATGCTCGCTGTCAGA

AAGTGTCACAGCAGCCTCAAAGGAGATGATATACAATTCATACATTTACAGTAACACAAT

GACGTTTAGAAATATCACTGCATTGCAGCATAGCATACCAACTAGCAATTCATTTGAAGG

GCACCTATTTTACCCCTTTTTCTAGATTTAAGATATGTTTTTCATAATGTGTTTGTAAAG

TTTCAGCTCAAAACATCAATCAGATTATTGGTTATAGCTTTCAGAGTCATGGAATCTTCT

GCTCTGAACACAATGTAGCTGTTTTTAATGCCTGTGCTTTTAATGCTAGTTCTCCCCGCC

CACCATTCACACGTGCCTGTTAAAGTGTGCCTTAATCTCCACCTCGGCTGCGTCAGATAA

Clone in Lane 10: **BLASTs to zebrafish Chr 9, location 1139078**

CCCACAATGTTGTTACATAGTTCACTCACAGACACACACGCACACACACAGCAAGCGCGT

TTAACTTTGCTGTGTTATTGCATGGCAAATGTAATAGGATACACATTAATATACACTGCT

CTATGGATATCTGAAGCTGATGTAACGTTCACAAACCGGGATTGATTTTAACATCTGCAA

ATAAAAAAAAAGGCTTATTGTCTTTATATCACTCCAGTATGACTGTAGAAACGCTATACC

TACACACAGTCTTTCCAAACAGCTTCCAAAAGATGATTTTTATCATAGGTGCCCTTTAAA

GATAGCTCAAAAAATGTTATAAACTTAGGATTACAAAATAAAACATTTCACAAAAATAAT

CTTCAAACAATTGATACAGTTTCAAAATTAAGACAAAAGTTTTGTTTTTATTAGTGGAAC

TGCACTATATTAGAATACCCTTTTTAATTTCTGTTCTTATTGATCTTATTTCGATATACG

AGCAGGTGCAGCCATTGGAATCTTTTTGGCTGAAGACTTCCGGTCTCATTCACTTCCATT

GATTTTTAGATGTTAAAAACGGCTCGTTATGCTGCTTTACGTTAAAAACTGATCTTTTCT

Clone in Lane 8: **BLASTs to zebrafish Chr 9, location 1136618**

GAAGGAAAATGAATGAATGAATAAATGTTATTAGGAATTTTATTAGAAATAAAATTAATG

GGATCTAATAATAAAATAAAACAATAGGAGGAAACAAATTTCATAGCATTCAAAAGAGTG

CAGTTTTTTTCTTGTTGTTGTTTTTTCTTGTTGTTGTTTTCATTTATTTCATTCTTTAGG

GTTGAAATATGGTTAAAATGTTTTTTTTTTTCTATTTCATTATTTCTTTACTTCAGTGTA

GGATCTTAATCACACACATGATCATTTCTAATGCAGTGACATTTATTTTTATTTACATAG

AAAACACATTTTTGGTCACACACAGATCTTCCGTTTCACACAACCTCCAGCATGAAACTC

TTTCATCTCTCTGAGTCCACTTTCAGCCTCTTGCAGTAGAGCAACCTTTGGCAAAAGCAA

TCTCGCGCAAGCGTTTCTGTCTCTGCTAACCAATTTTAACCTGCGCCTGAACTAGCAGCT

CTTTCCCAGGAGTCTTCGCTCTCTTTGCTGCAAGGAAGATTCTCTGGCTCCCATTTATCT

GTCTTTTTTTGTGTGACTGTCATTGAACAAGACTCATCTCTGTCTCTAG
